# Nanotechnology Approaches for Chloroplast Biotechnology Advancements

**DOI:** 10.3389/fpls.2021.691295

**Published:** 2021-07-26

**Authors:** Gregory M. Newkirk, Pedro de Allende, Robert E. Jinkerson, Juan Pablo Giraldo

**Affiliations:** ^1^Department of Botany and Plant Sciences, University of California, Riverside, Riverside, CA, United States; ^2^Department of Microbiology and Plant Pathology, University of California, Riverside, Riverside, CA, United States; ^3^Department of Chemical and Environmental Engineering, University of California, Riverside, Riverside, CA, United States

**Keywords:** plant nanobiotechnology, nano-enabled agriculture, chloroplast bioengineering, nanosensors, targeted delivery

## Abstract

Photosynthetic organisms are sources of sustainable foods, renewable biofuels, novel biopharmaceuticals, and next-generation biomaterials essential for modern society. Efforts to improve the yield, variety, and sustainability of products dependent on chloroplasts are limited by the need for biotechnological approaches for high-throughput chloroplast transformation, monitoring chloroplast function, and engineering photosynthesis across diverse plant species. The use of nanotechnology has emerged as a novel approach to overcome some of these limitations. Nanotechnology is enabling advances in the targeted delivery of chemicals and genetic elements to chloroplasts, nanosensors for chloroplast biomolecules, and nanotherapeutics for enhancing chloroplast performance. Nanotechnology-mediated delivery of DNA to the chloroplast has the potential to revolutionize chloroplast synthetic biology by allowing transgenes, or even synthesized DNA libraries, to be delivered to a variety of photosynthetic species. Crop yield improvements could be enabled by nanomaterials that enhance photosynthesis, increase tolerance to stresses, and act as nanosensors for biomolecules associated with chloroplast function. Engineering isolated chloroplasts through nanotechnology and synthetic biology approaches are leading to a new generation of plant-based biomaterials able to self-repair using abundant CO_2_ and water sources and are powered by renewable sunlight energy. Current knowledge gaps of nanotechnology-enabled approaches for chloroplast biotechnology include precise mechanisms for entry into plant cells and organelles, limited understanding about nanoparticle-based chloroplast transformations, and the translation of lab-based nanotechnology tools to the agricultural field with crop plants. Future research in chloroplast biotechnology mediated by the merging of synthetic biology and nanotechnology approaches can yield tools for precise control and monitoring of chloroplast function *in vivo* and *ex vivo* across diverse plant species, allowing increased plant productivity and turning plants into widely available sustainable technologies.

## Introduction

Chloroplast biotechnology has the potential to help alleviate the main challenges of this century by lowering renewable biofuels cost, increasing food production, and increasing productivity per plant. Currently, the cost of renewable energy through biofuels is not competitive against fossil fuels (Medipally et al., 2015; Mu et al., 2020; Lo et al., 2021; Scown et al., 2021). The current goal of the Bioenergy Technologies Office Advanced Algal Systems program within the Department of Energy is $2.5–3 gallon of gas equivalent for renewable algal biofuels by 2030, while current gasoline prices remain relatively low at $2.18 per gallon (BETO Publications, 2020; Fuel Prices, n.d.). Additionally, due to the rapidly growing world population, food production must increase by more than 50% in the coming decades with a more limited amount of arable and productive land and under a changing climate (Hatfield et al., 2011; Xu et al., 2013; Masson-Delmotte et al., 2018; Lowry et al., 2019). The “Green Revolution” in plant and molecular biology led to a significant increase in food productivity (Long et al., 2015). However, chloroplast biotechnology efforts toward increasing food production have been impaired by the inability to take full advantage of emergent research progress in synthetic biology and nanotechnology.

Stifling the ability to explore synthetic biology tools for the advancement of chloroplast biotechnology are the low chloroplast transformation rate, low number of species capable of having their chloroplast genomes transformed, and labor intensive culturing of calli – unorganized plant cells – and screening of phenotypes for homoplasmy. When compared to the rates of nuclear transformation within the same plant species, chloroplast transformation is a significant limitation. In-depth reviews of chloroplast transformation have been written by Day and Goldschmidt-Clermont (2011) and Bock (2015). Since its introduction, particle bombardment has been the standard method of chloroplast transformation across multiple plant species (Przibilla et al., 1991). This method attaches DNA to microparticles of gold or tungsten and, using a biolistic delivery system, propels the DNA-attached particle, *via* high-pressure helium gas, toward the plant cell. A significant downside to particle bombardment is that it requires specialized equipment and has a low transformation throughput. Accessibility, however, is limited to those plants for which protoplasts can be readily obtained (O’Neill et al., 1993). A more recent addition to the chloroplast transformation toolkit, a glass-bead vortex method has been demonstrated for green algae, but it does have lower rates when compared to particle bombardment (Economou et al., 2014; Wannathong et al., 2016). Despite these advances, relatively few plant species can have their chloroplast transformed with *Arabidopsis* being a very recent addition by Yu et al. in the Maliga Lab (Yu et al., 2017). Land plants routinely need species-specific bombardment procedures, vectors, and selectable markers. Increasing the number of species amenable to chloroplast transformation would have a significant research impact on broadening the number of crop plants that can be made more productive by bioengineering. A further hurdle is that any current chloroplast transformation method creates heterogenous chloroplast genomes, which must subsequently be driven to homoplasmy. Chloroplast genome replication, through cell division, is one way of producing and confirming a homogenous chloroplast genome. However, continuous calli culturing is a laborious and tedious manual process. Chloroplast transformation problems could be alleviated by a biomolecule delivery chassis that targets specific germline or meristematic plant cells and removes the tissue culture bottleneck. The benefits of a universal chloroplast transformation tool for diverse plant species could improve research in plant biology and have significant impacts on agriculture, the biopharmaceutical industry, and sustainable materials.

Advances in chloroplast biotechnology have broader impacts on medicine, fuel, food, bioplastics, and chemicals and may open new frontiers of crop development (Maliga and Bock, 2011). These chloroplast products become compartmentalized, which means they have unique abilities to produce advanced biopharmaceuticals like cancer-killing immunotoxins that would normally kill eukaryotic cells (Tran et al., 2013). Also, algal chloroplasts can produce high-value proteins like human growth hormones (Wannathong et al., 2016). Several reviews of biopharmaceuticals capable of being made within chloroplasts include Adem et al. (2017) and Dyo and Purton (2018). Additionally, chloroplasts can produce renewable fuel that is environmentally sustainable by utilizing carbon within the atmosphere rather than ecological carbon sinks (Medipally et al., 2015). A significant advantage of algae biofuels is that the biodiesel produced can work with existing gas infrastructure with slight modifications. The benefits of algal biofuels from advances in synthetic biology have been reviewed by Georgianna and Mayfield (2012). Chloroplast biotechnology advances also are leading to improvements in food crop productivity (Parry et al., 2013) of algae-based food products (Dawczynski et al., 2007). There are new frontiers of materials made from non-petroleum-based foam, where bioplastics are being used to make algae-based products from the starch made within chloroplasts (Mathiot et al., 2019). Through synthetic biology and the addition of artificial intelligence algorithms *via* deep learning, there could be even more opportunities for novel chemicals and crop improvements (Wang et al., 2020). To fulfill these chloroplast biotechnology breakthroughs, knowledge gaps in our current understanding of delivering synthetic biology tools must be addressed and molecular biology tools developed to be universal and more efficient.

Nanotechnology is providing tools to enable plant biology researchers for a better understanding of chloroplast molecular biology and genetics, by offering modular delivery chassis for chemicals and biomolecules, nanosensors, and nanotherapeutics that are customizable with targeted and controlled capabilities. Nanomaterials are particles within a size range of 1–100 nanometer scale and varying shapes, aspect ratios, charge, and surface chemistry. These nanoparticles can also be made up of diverse materials for biological applications including silica, gold, carbon, and polymers. Nanoparticles can be coated or loaded with biomolecules for delivery of cargo that can be targeted to plant cells and organelles, such as chloroplasts, by modifying their size and charge (Avellan et al., 2019; Hu et al., 2020) and biorecognition coatings (Santana et al., 2020). For example, single-walled carbon nanotubes (SWCNTs) can be coated with a single-stranded DNA for delivery to chloroplasts (Giraldo et al., 2014) with polyethylenimine for an overall positive charge to facilitate binding and release of plasmid DNA into the nucleus of a mature land plant (Demirer et al., 2019b) or with chitosan for the delivery and plasmid DNA to the chloroplast of a mature land plant (Kwak et al., 2019). Nanoparticles can also be fabricated with fluorescent properties such as carbon nanotubes and quantum dots for research on plant signaling, stress communication, and environmental monitoring (Giraldo et al., 2019). While knowledge of nanoparticle interactions with plants has increased in technological prowess, research into studying and engineering plants with nanomaterials are still in infancy.

**Figure 1:**
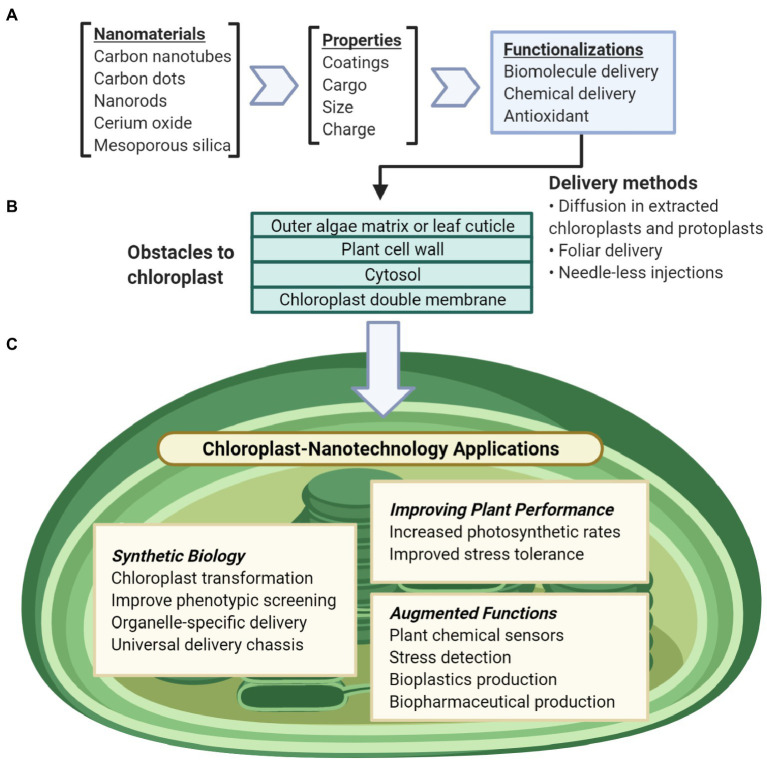
Overview of nanotechnology approaches for chloroplast biotechnology. **(A)** Nanomaterial properties can be modularly tuned for a variety of functions including biomolecule and chemical delivery, biosensors, antioxidants. **(B)** Nanomaterials can be delivered to chloroplasts in liquid suspensions by passive intake without mechanical aid or through needleless-syringes and foliar spray. To reach the chloroplast, these particles pass through obstacles of the plant cell including the outer leaf cuticle or the glycoprotein-rich extracellular matrix of algae, the plant cell wall, the plant cell membrane, the cell cytosol, and lastly the chloroplast double membranes. **(C)** Nanotechnology applications for understanding and engineering chloroplasts include synthetic biology research, improving chloroplast function, or enabling non-native abilities for chloroplasts.

This review focuses on nanotechnology uses that advance our understanding of chloroplast biotechnology (Figure 1). We discuss the current knowledge of the interactions between chloroplasts and nanomaterials, how plastid synthetic biology can synergize with nanotechnology approaches, nanomaterials’ impact on crop performance monitoring and improvement, and how nanotechnology can turn chloroplasts into manufacturing technologies.

## Chloroplast-Nanoparticle Interactions

Nanoparticle interactions with chloroplasts for biotechnology applications have been researched with isolated chloroplasts (Wong et al., 2016), plant protoplasts (Lew et al., 2018), and in leaves of land plants (Hu et al., 2020), but knowledge gaps remain on how nanomaterial properties, such as size, charge, hydrophobicity, and plant biomolecule coatings and coronas, influence interactions with land plants and green algae biosurfaces including the plant cuticle and cell wall and outer algae matrix, respectively. Although recent studies have improved our understanding of translocation of nanoparticles through chloroplast galactolipid-based membranes, how nanoparticle and membrane physical and chemical properties impact uptake into chloroplasts is not well understood.

**Figure 2:**
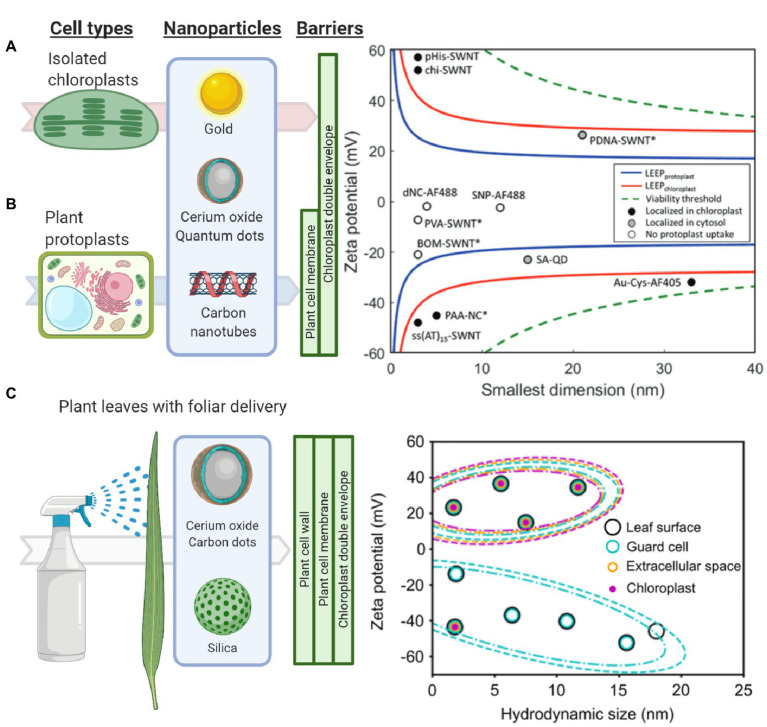
Understanding and modeling nanoparticle-chloroplast interactions. **(A)** The lipid exchange envelope penetration (LEEP) model was developed on isolated plant chloroplasts. It predicts that highly charged nanoparticles localize within chloroplasts while more neutrally charged nanoparticles are unable to enter these organelles (Reprinted with permission from Wong et al., 2016). **(B)** Similarly, the LEEP model for isolated plant protoplasts that includes a plant cell membrane as a barrier, predicts that nanomaterial charge magnitude determines whether particles enter plant protoplasts or localize in the cytosol or chloroplasts (Reprinted with permission from Lew et al., 2018). **(C)** Systematic studies of foliar delivery of nanoparticles of various sizes and charges *in planta* indicated that there is a size limit for uptake in leaf cells in which highly positively charged nanoparticles were more efficiently delivered into these organelles (Reprinted with permission from Hu et al., 2020).

Plant cell and organelle biosurfaces represent obstacles for delivering nanoparticles with their cargo into chloroplasts (Figure 2). Current standard particle delivery methods, such as particle bombardment, rely on pressure and force to deliver microcarriers to the chloroplast genome (Economou et al., 2014). More recently, nanomaterials have been delivered to chloroplasts by spontaneous penetration of lipid membranes *via* diffusion *in vitro*, leaf infiltration using a needleless syringe, and topical foliar delivery mediated by surfactants (Giraldo et al., 2014; Wong et al., 2016; Lew et al., 2018; Hu et al., 2020). The main barriers for entry into the chloroplast genome that nanoparticles must overcome are the plant cell wall, the plant cell membrane, the cytosol, and the chloroplast double membrane; in algae, there can also be an outer epilithic algal matrix (Kramer et al., 2014). Each of these plant biosurfaces represents various physical and chemical barriers that can limit nanoparticle uptake by size, charge, hydrophobicity, and other properties.

The plant cell wall comprises pectin, cross-linking glycan, and cellulose microfibrils (Barros et al., 2015) and is the first significant barrier to entry into the plant cell. The role of plant cell wall pore size, charge, and hydrophobicity have in limiting nanoparticle entry into cells has been recently reported but is not well understood. Nanoparticles up to 18 nm were capable of permeating cotton leaf cells, while nanoparticles larger than 8 nm could not permeate the maize leaf cells (Hu et al., 2020). This study, based on high spatial and temporal resolution confocal fluorescence microscopy, suggests that hydrophilic nanoparticles with a positive charge and less than 20 or 10 nm depending on plant type and leaf anatomy are more efficiently delivered into plant cells and chloroplasts. However, other studies have observed amphiphilic nanoparticles up to 40 nm to translocate across leaf cells and into other plant organs (Avellan et al., 2019). Additionally, studies of poly- and mono-dispersed poly(lactic-co-glycolic) acid nanoparticles have reported that the cell wall inhibits uptake in grapevine cells over 50 nm while the plasma membrane is permeable from 500 to 600 nm with the same nanoparticles (Palocci et al., 2017).

The cell membrane, which is a lipid bilayer composed of phospholipids, carbohydrates, and proteins, represents another barrier of entry into plant cells. Highly charged nanoparticles have been reported to cross both the plasma membrane and chloroplast envelopes (Giraldo et al., 2014; Wong et al., 2016; Lew et al., 2018). Passive penetration rather than energy-dependent endocytosis is hypothesized as the mechanism for nanoparticle uptake. The lipid exchange envelope penetration (LEEP) model proposes a disruption of the lipid bilayer by the ionic cloud surrounding nanoparticles (Figure 2B; Lew et al., 2018). Modeling studies of nanoparticle uptake by chloroplasts highlight the importance of nanoparticle charge. However, these models need to incorporate a variety of biosurfaces in plants such as plant cell wall, where nanoparticles encounter *in planta*. Furthermore, nanoparticles with varying hydrophobicities and biomolecules coatings and coronas have not been accounted for in the modeling efforts of chloroplast nanoparticle interactions. Recent evidence suggests that nanoparticles coated with a chloroplast guiding peptide do not require the high charge predicted by the LEEP model for targeting chloroplasts at high levels of more than 75% in *Arabidopsis* leaf mesophyll cells (Santana et al., 2020).

After crossing the cell wall and membrane, nanoparticles must then pass through the cytosol, containing a variety of different biomolecules, including proteins. Nanoparticles passing through the cytosol are expected to be coated with biomolecule coronas, but this is poorly understood within plants. Recently, Prakash and Deswal (2020) demonstrated that gold nanoparticles interfaced with plant extracts from *Brassica juncea* formed protein coronas increasing the nanoparticle surface charge by approximately 30% after 36 h of interaction. Mass spectrometry showed that 27% of the hard corona formation around the gold nanoparticle comes from the plant energy-yielding pathways including glycolysis, photosynthesis, and ATP synthesis (Prakash and Deswal, 2020). In comparison, a study on nanoparticle coronas with human plasma highlights that irrespective of the nanoparticle material, the coronas formed were dependent on size and surface engineering (Lundqvist et al., 2008). Research performed in mouse models reports that the wild-type *Tobacco mosaic virus* had a higher accumulation of proteins than synthetic nanoparticles, promoting faster clearance from the body (Pitek et al., 2016). This study also found that the choice of targeting ligand and surface engineering, e.g., coatings, can drastically alter the distribution and biocompatibility of the nanoparticles in living systems. These studies in non-plant systems indicate that protein, lipid, and carbohydrate coronas should be crucial to tune interactions with plant cells and organelles.

The last obstacles to reaching the chloroplast are its double lipid bilayers, referred to as the chloroplast membranes. The chloroplast membranes are formed by galactolipids and are highly dynamic (Block et al., 2007). Chemical interactions of nanomaterials with phospholipid-based membranes of eukaryotic cells have been thoroughly studied (Sanchez et al., 2012; Wu et al., 2013; Wang et al., 2016; Lew et al., 2018). However, there are no studies of nanomaterial interactions with the galactolipid-based membranes that form the majority of the chloroplast envelopes. Highly positively or negatively charged nanoparticles interact with the exposed lipids, allowing diffusion and eventual kinetic trapping into isolated chloroplasts without mechanical aid (Figure 2A; Wong et al., 2016). These nanoparticles can be larger than chloroplast porin’s diameter of 2.5–3 nm, and channel proteins, including mechanosensitive channels, have the largest diameter in chloroplast membranes (Ganesan et al., 2018). High and low aspect ratio nanomaterials, such as carbon nanotubes and carbon dots, respectively, are capable of penetrating plant cells and chloroplasts with high efficiency (Giraldo et al., 2014; Wong et al., 2016; Hu et al., 2020; Santana et al., 2020). However, the role of nanomaterial aspect ratio on entry into cells and chloroplasts has not been systematically explored with nanomaterials having precise control of aspect ratios. Gold, silica, and polymer nanostructures could aid in understanding the role of nanoparticle aspect ratio on interactions with chloroplast envelopes.

## Nanotechnology Cargo Delivery Approaches to Enable Chloroplast Synthetic Biology

**Figure 3:**
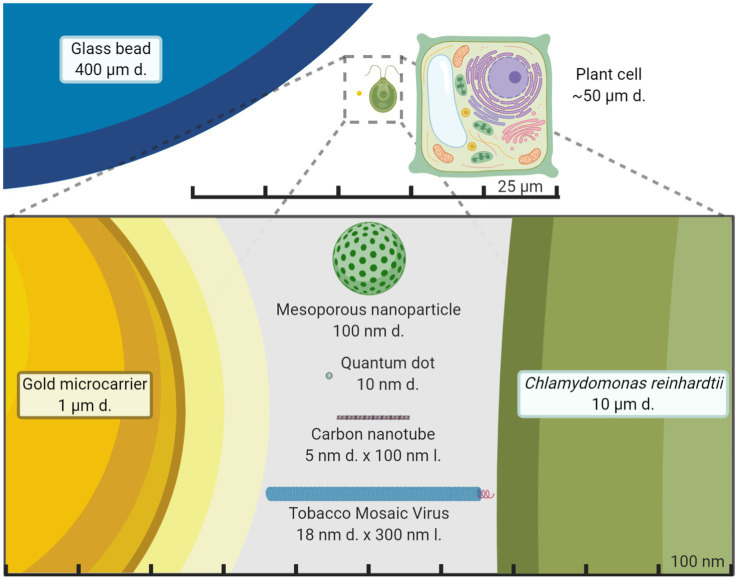
Size comparison of nanomaterials to other exogenous biomolecule delivery systems. Average plant cell and the green alga *Chlamydomonas reinhardtii* size are compared to chloroplast transformation carriers in the micrometer and nanometer scale. Gold microcarriers are standardly used in chloroplast transformations of both land plants and green algae through particle bombardment while the glass beads are used in a vortex-based protocol for *C. reinhardtii*. It becomes starkly apparent just how smaller nanoparticles are compared to standard microcarriers and potentially less disruptive for plant cells. The figure is made to scale.

Developing a universal and efficient chassis for biomolecule delivery into chloroplasts may unleash synthetic biology research progress into novel photosynthetic organisms, their molecular pathways and enable high-value biomolecule production. The advanced regulatory and expression logic systems constructed through synthetic biology are stymied by the inability to deliver biomolecules to chloroplasts. Ideally, this delivery chassis would cause little to no toxicity to the organism and have the ability to carry a variety of biomolecules (Vazquez-Vilar et al., 2018). Current approaches to deliver DNA to chloroplasts through force *via* particle bombardment work for a small number of organisms – nine species are shown with stable and reproducible plastid transformation (Bock, 2015) plus recently *Arabidopsis thaliana* (Yu et al., 2017) – but cannot be targeted to specific organelles. The standard gold or tungsten microcarriers used for chloroplast transformation in the gene gun system are 0.6–1.6 μm in diameter (Figure 3). These microcarriers have coatings that are not fully customizable, cannot be directed to specific organelles, or used without forced mechanical aid. Despite these limitations of current microcarriers and low transformation efficiency, synthetic biology has made enormous strides in plant biology research.

Despite being a relatively new field of research, synthetic biology has enabled the discovery of multiple new chemicals, exploration of advanced protein expression regulation, and production of novel high-value proteins like biopharmaceuticals within chloroplasts. These advancements in chloroplast biotechnology have been discussed in seminal reviews (Bock, 2015; Boehm and Bock, 2019). New research that is enabling chloroplast biotechnology includes the ability to monitor the expression of proteins *in vivo* through a luciferase reporter (Matsuo et al., 2006), gene activation can be enabled through a site-specific recombinase (Tungsuchat-Huang et al., 2011), a synthetic riboswitch (Verhounig et al., 2010), and metabolic pathway engineering is possible through synthetic multigene operons (Lu et al., 2013). These approaches may, in the future, be used in combination with nanotechnology approaches within diverse wild-type plants, for which currently there are no transformation and genome modification protocols available. Containing these new molecular and genetic regulatory mechanisms and proteins are possible through chloroplast biotechnology. As shown in the green algae *Chlamydomonas reinhardtii*, codon reassignment allows an additional avenue for biocontainment (Young and Purton, 2016). Biocontainment within chloroplasts may allow researchers to rapidly and specifically produce proteins within wild-type strains at specific time periods for a better understanding of nuclear-chloroplast protein expression and regulation. In addition, synthetic biology may be further enabled by a chloroplast transformation with large mutant libraries for the entire plastid genome. Facile *in vivo* assembly of chloroplast transformation vectors have been developed for plastid engineering (Wu et al., 2017a). The first fully exogenous plastid transformation has been completed in *C. reinhardtii* (O’Neill et al., 2012). The *in situ* ability of nanotechnology DNA delivery may enable new directed evolution approaches to screen large mutant libraries. Synthetic biology has made strides in research in a short amount of time, and new research done in nanotechnology may help to bolster it into new plant species.

**Figure 4:**
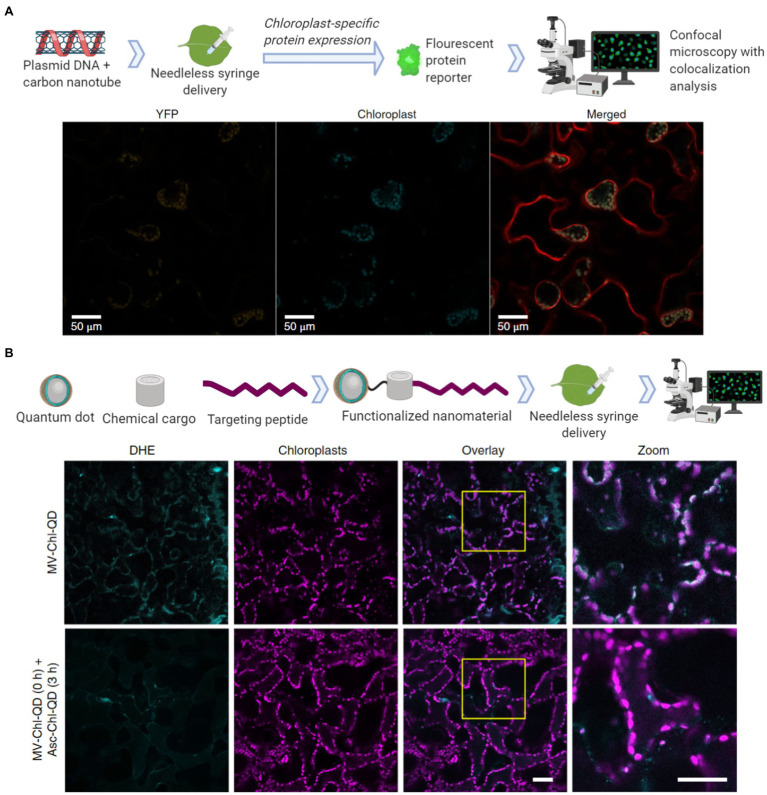
Nanomaterial mediated delivery of DNA and chemical cargoes to chloroplasts. **(A)** Single walled carbon nanotubes coated with chitosan carry plasmid DNA to chloroplasts. The nanomaterials are infiltrated into leaf mesophyll cells with a needless syringe. Confocal microscopy was then used for colocalization analysis 2–3 days post-infiltration by measuring the chloroplast-specific fluorescent protein (YFP) expressed within the mesophyll cells of tobacco plants (Material from Kwak et al., 2019). **(B)** Quantum dots with molecular baskets target the delivery of a chemical cargo to chloroplasts guided by a peptide recognition motif (Chl-QD). These functionalized nanomaterials were then loaded with methyl viologen (paraquat; MV-Chl-QD) to generate superoxide anion within chloroplasts or ascorbic acid (Asc-Chl-QD) to scavenge the superoxide anion. By monitoring reactive oxygen species (ROS) through the DHE dye using confocal microscopy, the targeted nanomaterials were shown to specifically induce and scavenge ROS *in vivo* in chloroplasts. Scale bar, 40 μM (Material from Santana et al., 2020).

Nanotechnology approaches are allowing the genetic modification for the expression of proteins and the specific delivery of cargoes to chloroplasts in wild-type plants. Chloroplast transformations currently must be performed with somatic or embryonic plant callus material and must be screened for heterogeneity in their genomes. This callus culturing stage requires manual labor and lengthy growth periods. New nanotechnology approaches are focusing on using mature land plants for the expression of exogenous DNA, which in turn may lead to the development of chloroplast transformations without calli culturing through targeted delivery into germline or meristematic tissues. Nuclear expression of exogenous DNA mediated by SWCNT has been assessed with a green fluorescent protein (Demirer et al., 2019a). Interestingly, the nuclear genomes seemed to not have been transformed as the incorporation of the exogenous DNA was not observed. A yellow fluorescent protein has also been transiently expressed from chloroplasts in mature *Eruca sativa*, *Nasturtium officinale*, *Nicotiana tabacum*, and *Spinacia oleracea* plants through SWCNT mediated delivery of exogenous plasmid DNA (Figure 4A; Kwak et al., 2019). One major advantage of nanoparticles is the ability to functionalize them with biomolecules for targeted and controlled delivery. A chloroplast targeting peptide allowed quantum dots to selectively target these organelles and to deliver chemical cargoes (Figure 4B; Santana et al., 2020). These nanotechnology advances in biomolecule delivery can act as promising tools for plant biology research and widespread use in crop biotechnology.

## Crop Improvements Through Chloroplast Nanobiotechnology

Agriculture demands more precise monitoring of plant health, increasing crop productivity, and efficiently delivering agrochemicals with lessening amounts of harmful environmental runoff. Chloroplasts are sites of photosynthesis, assimilation of nutrients, including nitrogen and phosphorus (Merchant et al., 2006; Carmo-Silva et al., 2015), and function as signaling organelles involved in plant stress responses (Van Aken et al., 2016; Su et al., 2019). More precise monitoring and improvement of photosynthesis, nutrient delivery to the sites of assimilation, stress responses, and plant health would allow higher crop yields. Some of these needs were met in the “Green Revolution” with molecular biology and genetics advancements that allowed higher crop productivity. However, chloroplast transformation-based approaches have not been reproducibly developed in most crops that feed the world (Bock, 2015).

**Figure 5:**
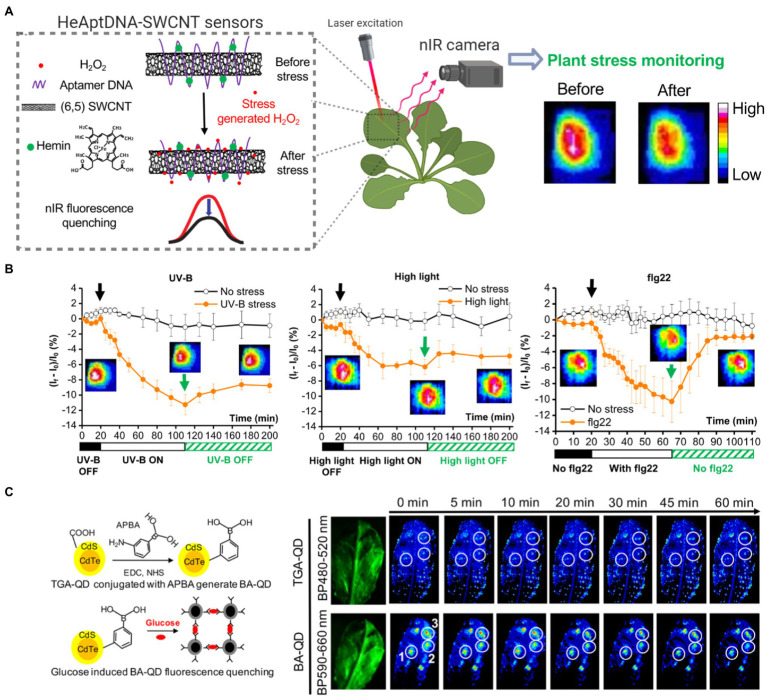
Plant health monitoring by nanotechnology-based sensors. **(A)** Single-walled carbon nanotubes (SWCNTs) can be functionalized with aptamer DNA and hemins to detect H_2_O_2_, a signaling molecule associated with plant stress. SWCNT sensors report H_2_O_2_ in real-time by quenching in NIR fluorescence intensity The nanosensor fluorescence changes are monitored by a NIR imaging before and after the stress event. This standoff detection *via* NIR imaging can report stress events within the plant physiological range of H_2_O_2_ (10–100 μm; Wu et al., 2020). **(B)** SWCNT-based nanosensors allow early detection of stresses from UV-B, high light, and a pathogen-related peptide (flg22; Reprinted with permission from Wu et al., 2020). **(C)** Boronic acid-coated quantum dots (BA-QDs) can act as glucose sensors, a principal product of chloroplast photosynthesis. Standoff glucose detection of *A. thaliana* is enabled by nanosensors excited through UV light and imaged with a Raspberry Pi camera (Reprinted with permission from Li et al., 2018). Thioglycolic acid-coated quantum dots (TGA-QD) act as internal controls that do not respond to glucose.

Recent advances in nanosensors research may allow nanotechnology-based devices that monitor plant’s health in real-time before detrimental symptoms occur. A full review of this topic discusses nanotechnology approaches for smart plant sensors (Giraldo et al., 2019), including nanosensors for monitoring plant health, detecting molecules related to photosynthesis, and reporting chemicals in the environment to electronic imaging devices already in use in phenotyping and agricultural operations. Current standard technologies that monitor plant function, stress, and photosynthesis rely on remote sensing tools to measure chlorophyll fluorescence or gas analyzers to quantify CO_2_ assimilation (Pérez-Bueno et al., 2019). Recently, carbon nanotubes were functionalized to sense H_2_O_2_, a key signaling molecule generated by chloroplasts and associated with plant stress (Wu et al., 2020). The H_2_O_2_ was monitored in real-time and within the plant physiological range through a near-infrared camera (Figures 5A,B). Multiplexed sensing of several plants signaling molecules associated with plant health, such as NO, glucose, and Ca^2+,^ among others, could allow for both monitoring plant stress status and identification of types of stress experienced. New research in nanotechnology has demonstrated the ability to use fluorescent quantum dots to monitor glucose, a direct product of chloroplast photosynthesis, through a Raspberry Pi camera in laboratory conditions in wild-type *Arabidopsis* plants (Figure 5C; Li et al., 2018). Previous approaches were only able to monitor glucose through genetically modified plant model systems. Plants embedded with nanosensors can also be engineered into environmental sensors for chemicals in groundwater with the use of remote near-infrared cameras. These plant nanosensors can detect small amounts of molecules in the environment such as those present in explosives (Wong et al., 2017). Although carbon nanotubes and quantum dots raise environmental toxicity concerns, improved knowledge in plant-nanoparticle interactions is leading to more precise control of the spatial and temporal distribution of nanomaterials in plant organs, such as leaves, for reducing exposure to humans and the environment (Wang et al., 2008; Williams et al., 2014). Alternatively, sentinel plants with nanosensors may be deployed throughout an area to determine what other plants within that crop field are experiencing.

**Figure 6:**
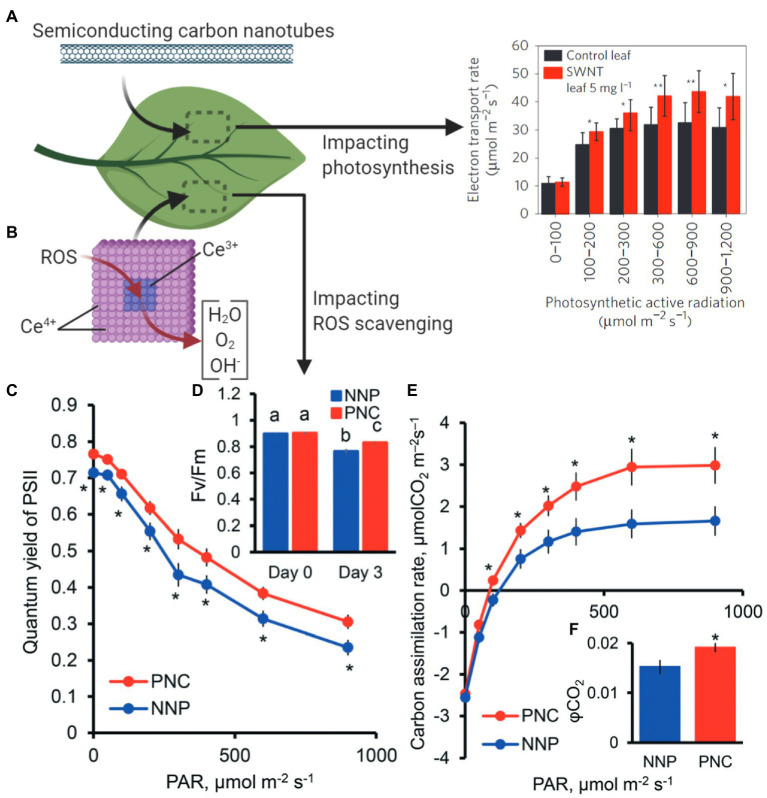
Nanotechnology approaches to improve plant photosynthesis. **(A)** SWCNTs interfaced with plant leaves increase chloroplast photosynthetic activity (Material from Giraldo et al., 2014). **(B)** Cerium oxide nanoparticles catalytically scavenge reactive oxygen species (ROS) in chloroplasts, resulting in enhanced light and carbon reactions of photosynthesis. Stressed plants with poly acrylic-coated cerium oxide nanoparticles (PNC) have higher **(C)** PSII quantum yields, **(D)** maximum PSII efficiency (Fv/Fm), **(E)** carbon assimilation rates, and **(F)** quantum yield of CO_2_ relative to controls without nanoparticles (NNP; Reproduced from Wu et al., 2018 with permission from the Royal Society of Chemistry). ^*^p < 0.05; ^**^p < 0.01. Different lower case letters mean significant differences at *p* < 0.05.

Bolstering chloroplast biotechnology through nanotechnology also may come through engineering photosynthesis in plants. Semiconducting SWCNTs have been shown to increase photosynthetic activity in mature plants (Figure 6A; Giraldo et al., 2014). The mechanisms of increased photosynthetic rates in land plants suggest that expanding the range of chloroplast pigment absorption to the near-infrared is a route for improving photosynthesis and is an avenue for new research. Nanotechnology approaches are enabling the improvement of wild-type plants without genetic modification by increasing their ability to scavenge reactive oxygen species (ROS) that are accumulated under abiotic and biotic stresses. Cerium oxide nanoparticles catalytically reduce hydroxyl radicals in *A. thaliana* leaves, a novel ability in plants (Figure 6B; Wu et al., 2018). This augmented hydroxyl radical scavenging capability improves plant stress tolerance by enhancing potassium mesophyll retention, which is a key trait associated with salt stress. Stressed plants interfaced with cerium oxide nanoparticles have higher carbon asssimilation rates, photosystem II quantum yields, and quantum efficiency of CO_2_ relative to controls without nanoparticles (Figures 6C–F; Wu et al., 2017b, 2018). Reducing ROS through nanomaterials is a promising mechanism for improving or maintaining plant productivity under stress environments in the field. While both of these examples are in the lab environment with a plant model species, they give an important stepping stone to future applications in the field in crop plant species.

## Future Research in Chloroplast Nanobiotechnology

Research into chloroplast biotechnology through nanotechnology approaches may take guidance from previous research breakthroughs in the biomedical field, lead to new discoveries through improved synthetic biology tools, and enable innovative ways of human-plant interactions, all while managing environmental impacts for applications in crops. Future chloroplast nanobiotechnology applications will range from targeted delivery of agrochemicals, plastid transformation and genome editing, nanosensors for monitoring signaling molecules, improvement of plant photosynthesis, and turning plants into biomanufacturing devices (Table 1).

**Table 1:** Strengths and weaknesses of nanotechnology approaches for chloroplast biotechnology advancements.

Chloroplast nanobiotechnology applications	Strengths	Weaknesses	Areas of improvement
Chemical delivery	Targeted delivery Less runoff into environment Improvement of agrochemical suspensions	Environmental impact of biocompatible nanomaterials for targeted delivery is unknown	Biodegradability studies of targeted nanomaterials Controlled chemical release
Gene delivery	Species independent Gene delivery without specialized equipment *In situ* gene delivery	Potential limitation of plasmid DNA size per nanoparticle Only transient expression shown Only proof of concept, with GFP expression	Stable plastid genome transformation enabled by nanomaterials Targeted gene delivery to chloroplasts for applied research Inducible expression of exogenous DNA Overcoming the bottleneck of selectable markers
Nanosensors	Real-time monitoring of plant signaling molecules High sensitivity down to single molecule level Do not photobleach Near infrared imaging Species independent	Available for only a few plant signaling molecules to date Most studies performed in laboratory conditions	Targeted sensor delivery to chloroplasts Multiplexed sensing of plant signaling molecules
Photosynthesis	Enhancement of light and carbon reactions of photosynthesis Protecting chloroplast photosynthetic machinery from oxidative stress	Environmental toxicity of some types of nanomaterials used to boost photosynthesis Most studies performed in laboratory conditions	Targeted delivery of nanoparticles to chloroplasts Develop more biocompatible and biodegradable nanomaterials that improve photosynthesis
Biomanufacturing	Use of plants as widely accessible, solar powered manufacturing technology Scalable, low cost manufacturing of biopharmaceuticals and bioplastics *in situ*	Research and development at very early stage	Proof of concept of turning plants into biomanufacturing devices using nanotechnology

To enable these future applications of nanotechnology for chloroplast biotechnology advancements will require improving our understanding of chloroplast-nanoparticle interactions. The role of nanomaterial hydrophobicity, aspect ratios, and biomolecule coatings on nanoparticle delivery to chloroplasts is not well understood. Hydrophobicity has been reported to play a role in altering the distribution of gold nanoparticles in plant leaves (Avellan et al., 2019). Recent studies have also explored how shapes of DNA nanostructures influence the delivery of siRNA-based gene silencing biomolecules in plant leaves (Zhang et al., 2020). Peptide coatings have recently been reported to more precisely guide nanoparticles to chloroplasts in plants (Santana et al., 2020). Future studies in these areas will be a significant step forward in understanding the underlying mechanisms of nanoparticle entry into plant cells and chloroplasts.

Chloroplast transformation is a limiting factor that, if alleviated, could fundamentally transform plant biotechnology research. Plant chloroplast transformation efficiencies are so low that researchers studying RuBisCO, the key protein responsible for CO_2_ assimilation during photosynthesis, have relied on bacterial systems instead of using plant or algae systems (Wilson et al., 2018). With efficient chloroplast transformation rates, large libraries could be used for the directed evolution of photosynthetic proteins; genome shuffling could be performed with the entire photosynthetic pathway; entire plastid genomes could be synthesized and mutated for increased photosynthetic abilities. For example, directed protein evolution is a strategy that takes advantage of large mutant libraries and yields mutants with a beneficial trait (Zhu et al., 2010; Sinha and Shukla, 2019). Recent research has enabled the simultaneous multiplexed synthesis of 7,000 synthetic genes for two essential genes in *Escherichia coli* (Plesa et al., 2018). While chloroplast transformation efficiencies may never achieve the efficiency of bacteria transformation, the most robust directed evolution experiment of a single chloroplast gene, RuBisCO’s *rbcL*, in *C. reinhardtii* was able to yield 80,000 library variants that were selected and screened across multiple chloroplast transformations (Zhu et al., 2010). While researching chloroplast transformation efficiencies, we found that there was a lack of standardization across research articles. Therefore, we are recommending reporting the following parameters to increase the scientific value and reproducibility of chloroplast transformations. Chloroplast transformations should be reported with raw data for (1) the amount and type of DNA used, (2) age and origin of calli, or cell count for algae, (3) amount of calli per plate bombarded, or algae cell count per transformation replicate, (4) transformants per replicate and total number before and after genetic screening, and (5) the transformation efficiency.

New synthetic biology applications and evolutionary strategies could help to bioengineer chloroplast genomes with, for example, improved efficiency through pathway engineering using robust mutant libraries and directed protein evolution. Synthetic genomics, i.e., the construction of chromosomes, is emerging in the last decade as an exciting frontier for minimizing genomes, constructing mosaic chromosomes of two or more species reengineering organelles (Coradini et al., 2020). Synthetic genomics approaches will be bolstered by nanoparticle gene delivery due to the ability to tune characteristics of the delivery chassis, deliver a wider array of genes for more applications at once, and allow gene delivery to precise organelles. In addition, nanotechnology approaches may be employed for CRISPR-Cas genetic engineering of plants (Demirer et al., 2021). With the chloroplast’s DNA repair mechanism nearly exclusively homology-driven, current approaches for plastid genetic engineering rely on delivering antibiotic markers with homologous arms for integration. However, a large problem with chloroplast biotechnology is the lack of strong selectable markers, like spectinomycin, necessary for marker excision through repeated rounds of transformation for chloroplast genetic engineering (Bock, 2015). In the future, CRISPR delivered by nanoparticles may enable new strategies of inducible silencing and increasing expression of exogenous DNA for pathway engineering that move beyond the bottleneck of strong selectable markers.

Nanomaterials can be used to deliver genes that encode proteins that act as sensors or the nanoparticle itself can be used as a sensor, and these approaches could lead to new applications in chloroplast biotechnology research. Through tunable characteristics and various types of nanoparticles, genes can be delivered that detect other proteins in wild-type plant species. For example, nanotechnology approaches for gene delivery of fluorescence resonance energy transfer (FRET) sensors to mature plants without previous genetic modification. In the future, multiplexed sensing of signaling molecules associated with chloroplast function may be possible. Currently, with *C. reinhardtii*, multiplexed stress-based imaging is possible through fluorescent-activated cell sorting (Béchet et al., 2017). In terms of applications to land plants, nanosensors already offer approaches to monitor chloroplast ROS, glucose, and nitric oxide (Giraldo et al., 2019). These plant signaling molecules may be able to be monitored simultaneously for the actuation of devices that promote plant health that are integrated into artificial intelligence deep learning algorithms.

Research into photosynthesis would be bolstered by nanotechnology approaches that allow targeted delivery of nanoparticles that manipulate chloroplast function. Biomolecule delivery of DNA or RNA (Wang et al., 2019) will expand research in the lab into chloroplasts of land plants that are not currently capable of being transformed. While nanotechnology approaches for plant research is a new field, nanoparticles have been used in mammalian systems to deliver biomolecules for the past decades (Shahiwala et al., 2007; Woodward et al., 2007). Their applications may give insights to future research directions in plants. CRISPR/Cas9 genome editing in mammalian cells through mRNA delivery has been demonstrated over the span of months (Liu et al., 2019). Applications in the field of nanoparticles for improving plant photosynthesis under stress will also require studies on environmental toxicity, the longevity of nanoparticles in the environment, and exposure of those nanoparticles to products for human consumption.

Plants and their chloroplasts potential are just beginning to be explored in terms of manufacturing of biopharmaceuticals, fuels, and materials. Plant chloroplasts within our homes may become 3D printers for high-value biopharmaceuticals (Maliga and Bock, 2011; Jin and Daniell, 2015). Polyhydroxybutyrate, a biodegradable polyester, can be made within the chloroplast and is being researched as a bioplastic (McQualter et al., 2016). Plants themselves could be used as a platform for self-repairing of infrastructure (Lu, 2020). A new class of materials made with extracted spinach chloroplasts stabilized with antioxidant cerium oxide nanoparticles can self-repair using glucose created from photosynthesis (Kwak et al., 2018). Algae and their chloroplasts enable a unique opportunity for renewable biofuels to take advantage of existing gasoline infrastructure and create jet fuel (Mayfield and Golden, 2015). In terms of legislation that is further enabling renewable algal biofuels, algae has officially been included in the latest 2018 Farm Bill in the United States, which enables algae agriculture to receive federal financial assistance for biomass cultivation, farm insurance, loans, carbon capture research and creates a new USDA Algae Agriculture Research Program (Conaway, 2018). These future applications for plants may fundamentally revolutionize our relationship with plants from providing food and materials to intricate partners that facilitate technology access to the world.

## Conclusion

Nanotechnology offers promising new approaches for some of the hardest challenges in chloroplast biotechnology research. Nanoparticle and plant cell interactions are still an emerging field that needs to be studied, but research has shown promising results in nanoparticles getting past plant cell barriers to their organelles. Knowledge gaps still exist in the exact mechanism of entry of nanoparticles into plant cells and chloroplast envelopes to determine the characteristics needed for a universal delivery cassette for biomolecules that would be applicable across diverse plant species. With current knowledge of plant-nanoparticle interactions, successful nanoparticle-based biomolecule delivery to chloroplasts has been possible. Using these targeted and controlled delivery technologies to bolster the number of applicable species or increase the efficiency of chloroplast transformations is yet to be seen. If increased chloroplast transformation efficiencies were to be realized, emerging synthetic biology-based strategies, such as directed protein evolution, may be able to be deployed within plastid genomes to unlock new potential in productivity and augmented manufacturing capabilities in chloroplasts. Additionally, nanomaterials have already been used to enable chloroplast biotechnology advancements such as sensing specific compounds, increasing photosynthetic rates, and decreasing stress-related molecules’ accumulation. Taken together, chloroplast biology and biotechnology research have challenges that can be uniquely addressed with nanotechnology approaches for increasing crop productivity and realizing the next generation of chloroplast-related biomanufacturing.

## Author Contributions

All authors listed have made a substantial, direct and intellectual contribution to the work, and approved it for publication.

## Conflict of Interest

The authors declare that the research was conducted in the absence of any commercial or financial relationships that could be construed as a potential conflict of interest.

## Publisher’s Note

All claims expressed in this article are solely those of the authors and do not necessarily represent those of their affiliated organizations, or those of the publisher, the editors and the reviewers. Any product that may be evaluated in this article, or claim that may be made by its manufacturer, is not guaranteed or endorsed by the publisher.
